# Distance and Angle Insensitive Radar-Based Multi-Human Posture Recognition Using Deep Learning

**DOI:** 10.3390/s24227250

**Published:** 2024-11-13

**Authors:** Sohaib Abdullah, Shahzad Ahmed, Chanwoo Choi, Sung Ho Cho

**Affiliations:** Department of Electronic Engineering, Hanyang University, Seoul 04763, Republic of Korea; engrsohaib79@hanyang.ac.kr (S.A.); shahzad1@hanyang.ac.kr (S.A.); choi231121@hanyang.ac.kr (C.C.)

**Keywords:** assisted living, human sensing, point cloud, FMCW radars, DenseNet

## Abstract

Human posture recognition has a wide range of applicability in the detective and preventive healthcare industry. Recognizing posture through frequency-modulated continuous wave (FMCW) radar poses a significant challenge as the human subject is static. Unlike existing radar-based studies, this study proposes a novel framework to extract the postures of two humans in close proximity using FMCW radar point cloud. With radar extracted range, velocity, and angle information, point clouds in the Cartesian domain are retrieved. Afterwards, unsupervised clustering is implemented to segregate the two humans, and finally a deep learning model named DenseNet is applied to classify the postures of both human subjects. Using four base postures (namely, standing, sitting on chair, sitting on floor, and lying down), ten posture combinations for two human scenarios are classified with an average accuracy of 96%. Additionally, using the centroid information of human clusters, an approach to detect and classify overlapping human participants is also introduced. Experiments with five posture combinations of two overlapping humans yielded an accuracy of above 96%. The proposed framework has the potential to offer a privacy-preserving preventive healthcare sensing platform for an elderly couple living alone.

## 1. Introduction

First-world societies are experiencing an increased life expectancy [1,2] coupled with a decrease in birth rates [3]. Elderly individuals often tend to reside either alone or with their spouse. Due to age-related challenges, individuals in this demographic often require continuous or at least frequent monitoring, and hospitals are already grappling with understaffing concerns [4]. In-home physical behavior monitoring systems hold a huge potential to prevent several aging-related medical emergencies. For instance, predictions related to falls in the elderly can be made by continuously observing their posture while performing daily life activities [5,6].

Previously, cameras have been widely utilized for posture classification; however, their use raises privacy concerns in home environments [7,8,9]. Radio transceivers such as radars provide a wireless posture monitoring solution with less privacy concerns. In addition to this, radar sensors are not directly affected by varying lighting conditions. Several authors have investigated the use of radar in the healthcare and assisted living domains [10,11]. Consequently, human posture classification using radar sensor has garnered a large amount of research attention lately [10,12,13].

Amongst radar technologies, a multi-input-multi-output (MIMO) frequency modulated continuous wave (FMCW) radar can simultaneously extract the range, angle, and the Doppler information of the target. Additionally, tasks related to multi-person posture classification insensitive of angle-of-arrival (AoA) requires a holistic scene representation followed by semantic segmentation. In such scenarios, rather than relying on the range-Doppler or angle-based information separately, the radar point cloud-based data representation schema can be adopted. In our work, FMCW radar point cloud data are subjected to the signal processing and machine learning pipeline to detect the posture of multiple persons.

### 1.1. Related Work

The task of multi-human detection has widely been studied for detection [14,15], tracking [16,17] people counting [18], and dynamic activity recognition tasks [19] using radar. Detection is relatively simple in comparison to the later tasks. For instance, Choi et al. [15] found multiple humans by computing peaks in distance axis. Sensing human vital signs after movement detection can be used to confirm human presence [20]. Similarly, multi-human tracking is often performed by clustering radar-returns followed by tracking with a Kalman filter [17]. Multi-human activity recognition has begun to establish footprints in the literature [19]. In comparison to static postures, dynamic activities provide a comparatively high amount of micro-Doppler information, making dynamic activities easier to classify in comparison to postures.

For static humans, radar point clouds can aid skeletal pose estimation as well as posture classification. Pose estimation is suited for rehabilitation and physical fitness-related applications [21,22,23]. However, without individual joint information, posture classification alone also has the potential to provide detective and persuasive healthcare solutions. Consequently, a considerable amount of work has been published surrounding daily life posture classification. One of the earliest attempts to classify human posture considered a pulsed radar to classify sitting, standing, and lying posture using forty different features [24]. For similar postures, the authors in reference [25] used a decision tree classifier driven by 33 features, and achieved an overall accuracy of 85%. Another study [26] extracted point cloud representations of six different postures through a novel framework and achieved a success rate of 54.6% by comparing the dimensions of the point cloud with original postures. However, all of these studies were focused on single human at a fixed distance and angle.

Recently, Zhao and co-workers [13] proposed an angle-insensitive posture recognition system based on point cloud data extracted using FMCW radar. The point cloud-based spectrograms images were used as input to the pre-trained AlexNet, which demonstrated over 87% accuracy. However, only a single human subject (and single distance) was considered for point cloud generation. Yang et al. [27] classified three postures at different distances and angles for a single human subject using only range information, which may not be very robust in real applications. Wu et al. [28] recognized three postures along with heart rate estimation in their study; however, only three postures and a single human subject were considered.

The authors in reference [29] recognized only asiting posture with five different poses using FMCW radar and a feature-based support vector machine (SVM), which provided 96% accuracy. Another study opted to employ a lightweight convolutional neural network (CNN) architecture on a voxelized point cloud to recognize sitting direction [30]. A recent work extracted vital signs and detected fall posture in a single human subject [31]. Another work classified seven postures of a walking pedestrian [32]. Similarly, De et al. [33] classified two postures and one activity, and focused mainly on comparing different classification techniques. Table 1 summarizes the related literature considering posture classification works intended for (elderly) healthcare.

As stated earlier, elderly individuals tend to either live alone or with their spouses. However, the prior literature suggests that the existing works consider only a single human subject for posture classification [10,12,13], overlooking the scenario of an independently living elderly couple. In addition, multiple distances and angles and overlapping human subjects have not been studied so far.

### 1.2. Our Work: Scope and Novelty

In this article, we propose a novel multi-human posture recognition framework using a millimeter-wave (mmWave) radar point cloud and deep learning architecture. An off-the-shelf (OTS) FMCW radar consisting of 12 transmitting and 16 receiving antennas is used in this study. Four base postures from two human subjects are captured using MIMO FMCW radar. Two humans collectively resulted in ten combinations of postures in each data capturing scenario. The captured radar-returns are converted into 3D point cloud data, and unsupervised clustering is used to separate the two targets. Once the postures of two humans are separated, a DenseNet based deep learning architecture is utilized for feature extraction and classification. The main contributions and novelty of our work are as follows:

1. To the best of our knowledge, this is the first study to estimate human postures from multiple (two) subjects using FMCW radar point cloud. An unsupervised clustering-based strategy to separate human targets is presented. Consequently, such systems can facilitate a detective and preventive healthcare system for elderly couple.
2. Additional experimentation with both humans present in a close proximity is also performed, allowing a partial overlap between the two human subjects. The distance between the clustering centroid of the two humans is evaluated to confirm the close proximity. In such a case, the first cluster is considered as the first human subject whereas the second and third clusters are combined together to reconstruct the posture of the second human subject. Although the point cloud was visually deteriorated, the deep learning algorithm was able to classify five posture combinations.

Deep learning-based approaches for radar-based activity and posture classification are on the rise [4,37]. In this work, the posture point clouds are exported as an image, followed by deep learning-based classification. CNN has been a dominant paradigm over the past decades. A CNN structure relies on multiple layers of convolution and max-pooling to learn features from image data, and ultimately, a soft-max layer converts feature vectors into class probability. Stacking several layers to form a complex network is a well-known approach in the literature.

Instead of linearly stacking convolutional layers, the formation of deep non-linearly stacked convolutional models was originally introduced in 2012 when a complex CNN structure termed as AlexNet outperformed the existing image classifiers [38]. Since then, several complex structures have been proposed [39,40,41]. This study utilizes DenseNet architecture [41] to classify radar-generated point cloud images of human postures. DenseNet offers an improved gradient flow and implicit regularization to mitigate vanishing gradients and overfitting issues, respectively. In addition to this, in comparison to competing architectures such as ResNet [42], DenseNet is computationally efficient and provides diversified features. DenseNet models have shown promising accuracy on point cloud data.

The rest of this paper is organized as follows: Section 2 presents the materials and methods; Section 3 defines the experimental setup and data collection scenarios; Section 4 presents the results and discussion; and finally, Section 5 concludes the paper.

## 2. Materials and Methods

This section provides a detailed overview of the proposed framework, consisting of radar signal acquisition and pre-processing followed by radar point cloud extraction and posture classification.

### 2.1. Signal Acquisition and Pre-Processing

**Figure 1:**
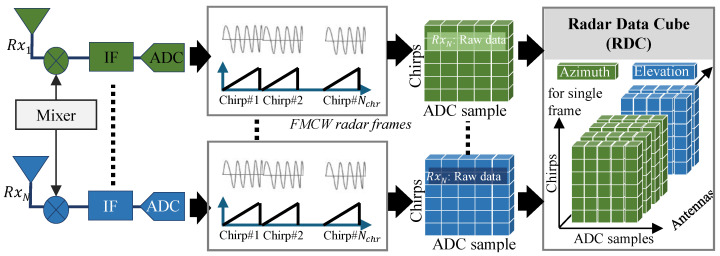
FMCW radar signal acquisition in MIMO configuration.

A FMCW radar frame consists of several chirps characterized by linearly increasing frequency signals (see Figure 1). The transmitted frame x(t) can be represented as

(1) $$x\left(t\right)=exp\{j2\pi \left(fct+\frac{B}{2T}t^{2}\right),0\leq t\leq T,$$

where $f_{c}$ is the initial frequency, *T* is the chirp time, and *B* is the bandwidth. The rate at which chirp ramps up is determined by slope *S* and chirp time $T_{c}$. A higher bandwidth is proportional to higher range resolution, which in turn corresponds to multiple reflections from the human body and higher point cloud density. The corresponding signal reflected from target present within the radar field-of-view (FOV) is collected at the receiver. The received signal $x_{r}$ is the time-delayed and attenuated version of a transmitted signal such that

(2) $$x_{r}\left(t\right)=exp\{j2\pi \left(fc\left(t-t_{d}\right)+\frac{B}{2T}{\left(t-t_{d}\right)}^{2}\right).$$

The term $t_{d}$ is time taken by the transmitted chirp to collide with the target and be received back at receiver antenna. Figure 1 illustrates the processing of the received signal, where the first step is to pass the signal through a mixer to acquire a low frequency signal termed as an intermediate frequency (IF) signal, which is expressed as

(3) $$IF\left(t\right)=exp\{j4\pi \left(\frac{BR}{cT}t+\frac{R}{\lambda }\right\}),$$

where λ is the wavelength of IF signal. For a MIMO radar, the transmitting and receiving antennas can be used sequentially through time-division multiplexing (TDM) to form a virtual antenna array. As expressed in Figure 1, the whole operation is repeated individually for each receiving channel (Rx). The IF signals of each Rx within a defined coherent processing interval (CPI) are sampled through an analogue-to-digital converter (ADC) and stored in a three-dimensional array known as a radar data cube (RDC). The size of the RDC array depends on the number of samples, number of chirps, and number of frames.

### 2.2. Point Cloud Generation

**Figure 2:**
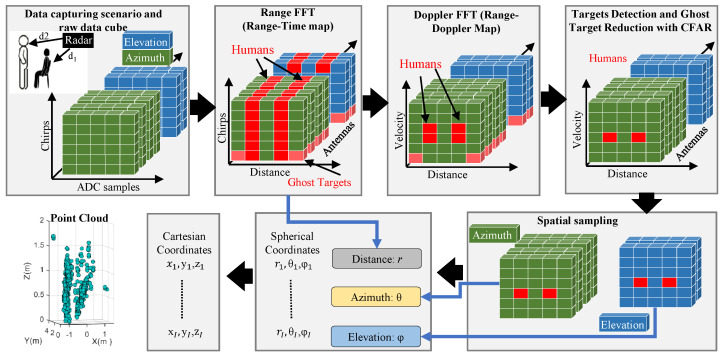
Conceptual point cloud generation framework with two persons present at different distances.

Figure 2 illustrates the point cloud generation framework from the raw data captured for a two-person scenario. The RDC is passed through a Fast Fourier Transform (FFT) computation block in a horizontal direction along the ADC samples. This FFT is often termed as range-FFT [43,44], and the peaks in range-FFT define the distance (*r*) of targets as illustrated in Figure 2. Next, another FFT (Doppler-FFT) is performed along the number of chirps in a frame, which yields the velocity (*v*) of the target. The Doppler-FFT requires the transmission of multiple chirps within a single frame.

In order to reduce ghost targets, a suitable target detection algorithm must be applied on the range-Doppler map to detect the human target in the noisy environment. Constant false alarm rate (CFAR) is a common choice for this purpose due to its ability to operate under variable noise floor [30,45,46]. We implemented cell-averaging smallest of-CFAR (CASO-CFAR) due to its ability to detect multiple adjacent targets in a radar FOV [47,48].

To extract the AoA, a third FFT along the receiving channels is performed individually at each target range-bin to calculate azimuth (θ) and elevation (ϕ) angles. In this way, *r*, θ, and ϕ collectively define the location of a single reflection from the human body in spherical coordinates, and the overall reflections for an arbitrary frame *f* will be

(4) $$P_{f}\left(r,\theta ,\phi \right)=\left\{\left(r_{i},\theta_{i},\phi_{i}\right),i=1,2,\ldots ,I\right\},$$

where $P_{f}$ represents the collection of target reflections detected by CFAR for the frame number *f*, and *I* is the total number of target reflections in a single frame. The velocity (*v*) conveys the motion characteristics; however, it can be ignored while dealing with static postures. The point cloud data in the Cartesian coordinates *x*, *y*, and *z* can be calculated using *r*, θ, and the below:

(5) $$P_{f}\left(x,y,z\right)=\left[\begin{array}{c} x_{i}\\ y_{i}\\ z_{i} \end{array}\right]=\left[\begin{array}{c} r_{i}sin\left(\theta_{i}\right)cos\left(\phi_{i}\right)\\ r_{i}cos\left(\theta_{i}\right)cos\left(\phi_{i}\right)\\ r_{i}sin\left(\phi_{i}\right) \end{array}\right],\;i=0,1,2,\ldots ,N_{fr}.$$

where the term $N_{fr}$ represents the total number of frames required to generate the posture point cloud. The point cloud from multiple frames is accumulated together to form *P*, which is further considered to extract the shape of a human present in the radar FOV:

(6) $$P=\left[\begin{array}{ccccc} p_{1} & p_{2} & p_{3} & \ldots & p_{N_{fr}} \end{array}\right].$$

Once the point clouds are formed, the spatial and temporal characteristics such as shape, position, and motion of a human target can be acquired in the point cloud domain. Temporal modeling requires clustering within one frame, whereas spatial modeling, such as for posture, requires frame aggregation [49].

### 2.3. Multiple Humans Detection with Unsupervised Clustering

The spatio-temporal information of human targets conveyed by the point cloud map *P* also contains noise points and outliers due to interference and multi-path effects [50]. In addition, multiple humans also form a cluster of co-located points, suggesting the use of an unsupervised clustering algorithm. Consequently, a clustering approach known as density-based spatial clustering of applications with noise (DBSCAN) is used to reject the noise and detach two human clusters at the same time. DBSCAN is a common preference in radar-based point cloud processing [51,52]. The outputs $C_{1}$, $C_{2}$, and $C_{3}$ of DBSCAN represent the three clusters of co-located points such that

(7) $$\left[C_{1},C_{2},C_{3}\right]=\mathrm{dbscan}\left(P\right),$$

and the cluster centroids $c_{1}$, $c_{2}$, and $c_{3}$, corresponding to the three clusters $C_{1}$, $C_{2}$, and $C_{3}$, are computed as

(8) $$\left[c_{1},c_{2},c_{3}\right]=\left[\frac{\sum C_{1}}{n},\frac{\sum C_{2}}{n},\frac{\sum C_{3}}{n}\right].$$

**Figure 3:**
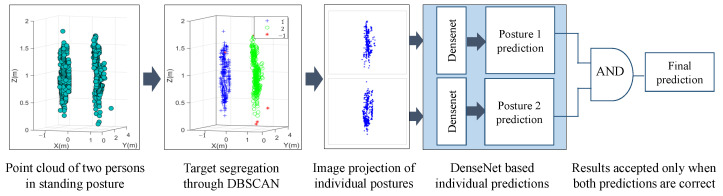
Overall strategy to transform raw point cloud into images followed by posture prediction.

Figure 3 briefly illustrates the methodology to separate the two human targets using DBSCAN. In non-overlapping cases, clusters $C_{1}$ and $C_{2}$ are designated as human 1 and human 2, respectively, and processed individually. The two postures are transformed into images, and a deep learning algorithm is applied individually to both the images. The two predictions are passed through an AND logic, where the overall prediction is considered as correct only if both the postures are detected correctly.

For the overlapping case, five posture combinations are considered, which are based on three individual postures: standing, sitting on a chair, and lying down. Sitting on the floor was not considered, since the available point cloud information was not enough to generate the corresponding point cloud. In overlapping cases, the centroids of each cluster are observed to judge whether the point clouds of the two participants are overlapping each other or not. If the distance between the three centroids is smaller than the adjusted threshold (th), an overlapping case is detected, and the third cluster is combined with second cluster to form a single image. On the other hand, if the distance between two clusters is higher than the th, the third cluster is ignored as noise.

The aforementioned clustering approach separates the two human subjects in the radar point cloud domains. As illustrated in Figure 3, the separated point clouds are further saved as two independent 2D images in the (*x*,*z*) Cartesian domains. The point cloud appeared to be symmetrical along the y-axis, which suggests that 2D *x*,*z* information can effectively represent the overall posture data. Subsequently, an image classifier can be trained to classify the independent posture of each human. Note that in single iteration, the postures of both the humans are classified individually, and the overall prediction is labeled as true only when both the predictions are correct.

### 2.4. Posture Classification Using Deep Learning

**Figure 4:**
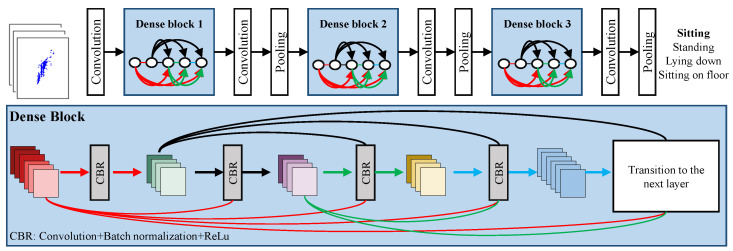
DenseNet architecture for postures classification.

As stated earlier, non-linearly structured convolutional networks have emerged as a promising solution in image recognition problems. In this study, several machine learning architectures were evaluated for radar-based posture recognition work, and the best performing architecture was adopted. The deep learning architecture considered in this study is shown in Figure 4. Along with convolution and pooling layers, the network comprises a complex block termed as a dense block, where all the dense block inputs and intermediate outputs are connected to form a dense connection. Each dense block perpetuates the same process, as illustrated in Figure 4. Subsequently, feature diversity is achieved for enhanced performance. Our network consists of three dense blocks for feature learning, followed by classification.

## 3. Experimental Setup

To validate the proposed two-human posture recognition framework, extensive experimentation is carried out at different distances and angles, along with different overlaps.

### 3.1. Experimental Configurations

**Figure 5:**
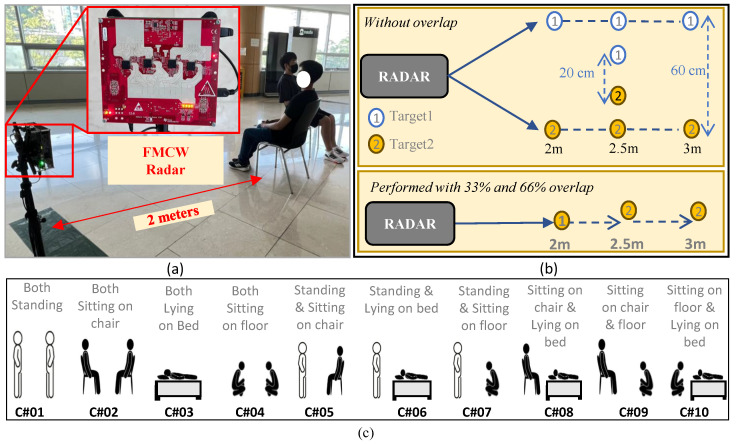
Experimental setup for data capture: (**a**) Two participants sitting in front of MIMO FMCW radar, (**b**) all the data capturing locations, and (**c**) ten possible combinations for non-overlap scenarios.

For data collection, we used mmWave radar, named MMWCAS-RF-EVM, manufactured by Texas Instruments (Dallas, TX, USA). This radar consisted of 12 transmitting and 16 receiving antennas. A total of 192 receiving channels were formed using TDM-MIMO, out of which 86 channels corresponded to the azimuth antenna array, and 4 channels corresponded to the elevation array. The parameters for configuration are presented in Table 2. A frame rate of 20 frames per second (FPS) was selected, while the total capture time for a single data sample was set to 1 s. One of the data capturing scenarios is depicted in Figure 5a, showing two participants sitting with 33% overlap in front of FMCW radar at a distance of approximately 2 m. As illustrated in Figure 5b, to confirm the robustness of proposed framework, the data were captured both in overlap and non-overlap scenarios. The resulting posture combinations for the two non-overlapping human case scenarios are illustrated in Figure 5c. Note that a single scenario was marked as correct recognition only if both the postures were classified correctly.

### 3.2. Participant Demographics

Five participants were involved in the data capture scenarios, with an average height and weight of 1.70 ± 0.38 m and 73.9 ± 8.6 kg, respectively. Given that the posture recognition experiments involved real human participants, an approval from the research ethics committee at Hanyang University, Seoul, South Korea was received prior to experimentation. The related Institutional Review Board (IRB) ID is HYU-2021-01-015. Additional consent was also gathered from the involved human volunteers. Lacking the availability of open-source datasets, radar-based research works have mainly been conducted by capturing data first. In this work, we used 70% of the data for training purposes, and the remaining 30% for test purposes. The dataset comprised a total of 900 images.

### 3.3. Data Collection for Non-Overlap Case

The data collection setup for the non-overlap cases is shown in Figure 5c. The two participants performed four different postures at different distances and angles. The considered postures were standing, sitting on a chair, sitting on the floor, and lying on a bed. This scenario resulted in ten posture combinations, as shown in Figure 5c. A total of 600 samples were collected for non-overlapping postures of the two participants.

### 3.4. Data Collection for Overlap Cases

In real-world scenarios, it is not necessarily true that both participants will be at different angles; instead, they might be at the same angle, i.e., one might be overlapping another. In such cases, the accuracy of any posture estimation system may drop, as partial information from one of the postures is missing. To assess the impact of different degrees of overlap, three postures named as standing, sitting on chair, and lying on bed were considered. The fourth posture, sitting on floor, was not considered, since no line of sight was available to generate the relevant point cloud. These three postures resulted in five posture combinations for two-human scenarios.

**Figure 6:**
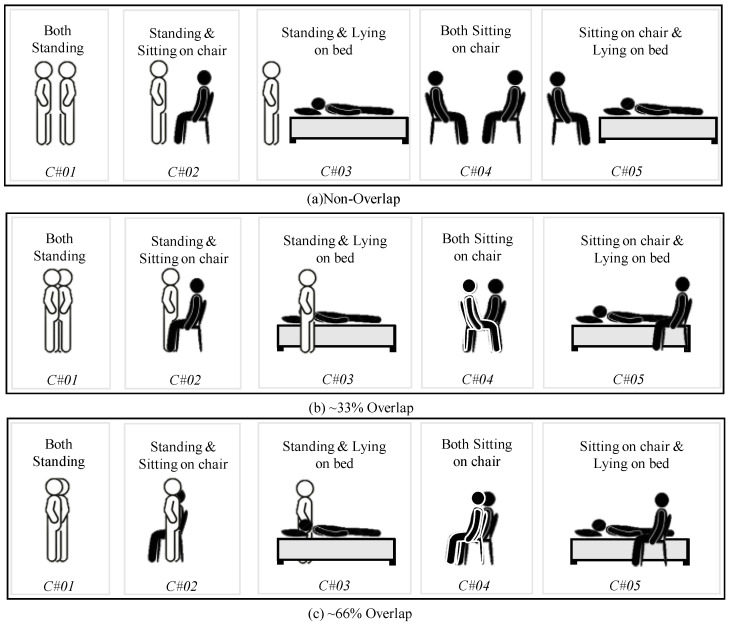
Posture combinations for non-overlap and overlap cases combined: (**a**) Non-overlap case with 5 posture combinations, (**b**) ≈33% overlap, and (**c**) ≈66% overlap.

The designed experimental setup is shown in Figure 6a–c. In the first case, although both participants were visible to radar, they were in close proximity, as shown in Figure 5b. For the second and third cases, one of the participants was completely visible to the radar, while overlapping approximately one-third (33%) and two-thirds (66%) of the other participant, respectively. The posture combinations for these three cases are depicted in Figure 6. For the combined non-overlap and overlap cases, 900 images were collected in total.

## 4. Results and Discussion

### 4.1. Point Cloud Visualization

**Figure 7:**
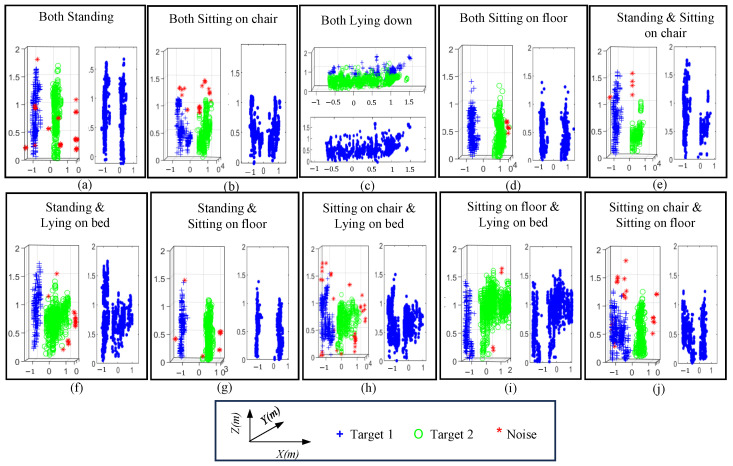
Point cloud of all the ten posture combinations: (**a**) Both standing, (**b**) both sitting, (**c**) both lying down, (**d**) both sitting on floor (**e**) standing and sitting, (**f**) standing and lying down (**g**) standing and sitting on floor, (**h**) sitting and lying down, (**i**) sitting on floor and lying down, and (**j**) sitting on chair and lying down.

Figure 7 shows the clustered point clouds and the corresponding images for all the ten posture combinations of two humans in a non-overlapping scenario. The blue, green, and red colors correspond to person one, person two, and noise artifacts, respectively. The DBSCAN approach effectively clustered each human present in the radar FOV while reducing the noise artifacts. For instance, Figure 7a shows the point clouds of persons standing in front of the radar. Similarly, Figure 7b shows the point clouds of standing and sitting human subjects.

**Figure 8:**
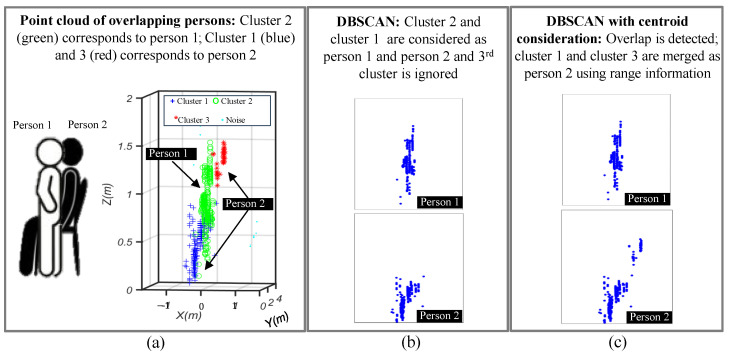
Radar point cloud for two overlapping human participants in standing and sitting on chair positions: (**a**) data collection environment and extracted point cloud, (**b**) raw DBSCAN, and (**c**) centroid-based overlap detection.

Radar data, represented as point clouds, are often beneficial for complex recognition tasks such as semantic segmentation and multiple object recognition. The point clouds shown in Figure 7 illustrate that once the point cloud of a human subject is exported as an image, computer vision approaches can effectively learn the features and extract the individual postures afterwards. The target separation results are illustrated in Figure 8 for convenience.

### 4.2. T-SNE Analysis of Opted Network

**Figure 9:**
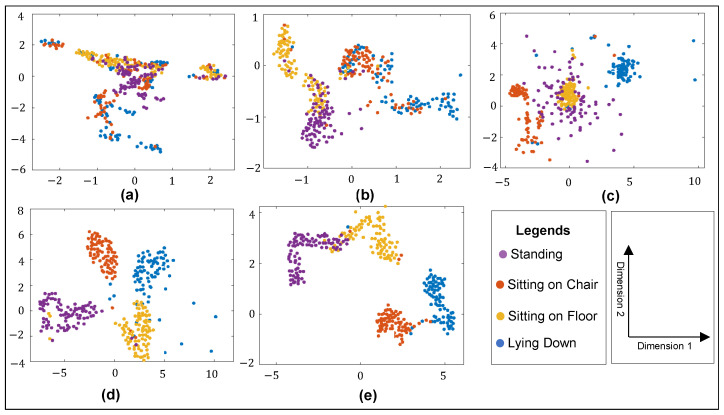
T-SNE analysis for (**a**) 7-layered CNN, (**b**) 16-layered CNN, (**c**) 25-layered CNN, (**d**) DenseNet, and (**e**) ShuffleNet.

The t-distributed stochastic neighbor embedding (t-SNE) analysis is a frequently used dimensionality reduction technique to visualize the complex dataset using low dimensional space. Prior to the accuracy evaluation of the opted deep learning model, features visualization at the end of the learned network can be performed to visualize the features distribution in lower dimensions. The performance of different networks in lower dimensions using t-SNE analysis is presented in Figure 9a–e, which illustrates that the inter-class separation for the learned features of ShuffleNet and DenseNet is higher than the other networks. Within these two networks, inter-class separation for DenseNet based features was the highest.

DenseNet has a wider feature distribution in comparison to the other four networks. Both DenseNet’s enhanced ability to learn the patterns present in the data and facilitate improved discrimination between different classes more effectively can be attributed to the presence of dense connections that each layer has with all the subsequent layers, allowing increased flow of feature maps within the network [53]. Next, the performance of the opted network is computed with multiple metrics.

### 4.3. Metrics for Performance Evaluation

Once all the point cloud images of all the postures were created, the dataset was labeled and 70% of the data was used for training, whereas the remaining 30% was used for test purposes. The dataset comprised a total of 900 images. Since the scope of classification spanned over multiple classes, we evaluated the performance of proposed framework through multiple metrics named as Precision, Specificity, Recall, F1-score, and the area under the curve (AUC). Precision and Specificity quantify the accuracy of the true prediction and false prediction of a particular class, respectively, in comparison to all the instances of that class. Recall measures all the actual true instances. In short, Precision measures the performance of a particular class to avoid false-positives for the positive class, Specificity measures the performance of a particular class to avoid for the negative class, and Recall measures the ability to reduce false-negatives. These terms can be expressed as,

(9) $$Precision=\frac{TP}{TP+FP}.$$

(10) $$Specificity=\frac{TN}{TN+FP}.$$

(11) $$Recall=\frac{TP}{TP+FN}.$$

In (9)–(11), TP, FP, TN, and FN represent true-positive, false-positive, true-negative, and false-negative, respectively. Precision and Recall collectively form the F1-score, which can be termed as a harmonic mean between the two quantities defined as,

(12) $$F1\text{-}Score=\frac{2\times Precision\times Recall}{Precision+Recall}.$$

The AUC value, which corresponds to the probability that the model ranks a randomly chosen negative instance lower than a positive instance, can be computed from the area under the receiver operating characteristics (ROC) curve by plotting recall or true positive rate (TPR) against the false positive rate (FPR) defined as

(13) $$FPR=\frac{FP}{FP+TN}.$$

### 4.4. Performance Evaluation for Non-Overlap Case

**Figure 10:**
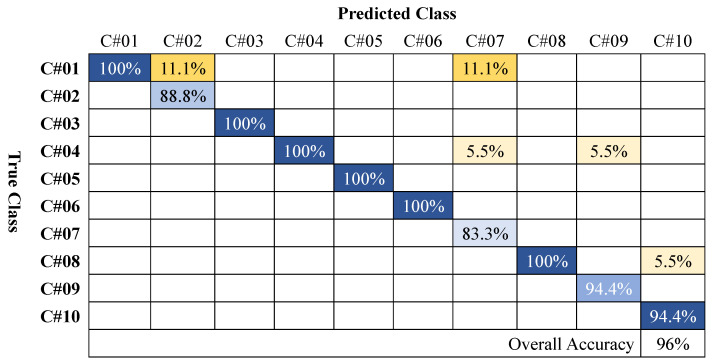
Confusion matrix for all the ten posture combinations in non-overlapping cases.

When both participants are posed in a non-overlapping fashion, complete postures can be retrieved using the radar point cloud, as illustrated in Figure 7. The corresponding confusion matrix for this case is shown in Figure 10. The proposed framework achieved a high accuracy of 96%. As stated earlier, the final prediction was considered as correct only if both the postures were identified correctly (see Figure 3). Figure 10 suggests that the sitting on ground posture was often mixed with the other posture, resulting in a higher error rate. The rest of the performance metrics are reported in Table 3.

### 4.5. Overall Accuracy Including Overlap and Non-Overlap Cases

The overall performance for the two overlapping and non-overlapping human subjects was evaluated using the data capturing scenario introduced earlier in Figure 6. The proposed framework first detected the overlap using the DBSCAN algorithm as illustrated in Figure 8. Since the DBSCAN clustering works on the principle of connected density, it tended to divide the occluded postures into two separate clusters as illustrated in Figure 8a. The point cloud of the first person is shown in green, whereas the point cloud of the second person consists of two clusters shown in blue and red. DBSCAN, without considering the centroid information to evaluate whether overlap occurred or not, may consider cluster 1 and cluster 2 as target 2 and target 1, and thus the upper body part of second person is ignored, as illustrated in Figure 8b. On the other hand, the centroid information-assisted DBSCAN clustering technique first detects an overlap if the centroids are present very near to each other. Subsequently, the two clusters whose centroids are present at same range are merged together to provide additional information about the occluded posture, as shown in Figure 8c. This additional information further enhances the classification results in overlapping cases.

The performance evaluation for the two overlapping human subjects is summarized in Table 4. For all of the cases, considerable accuracy is observed; however, a decreasing trend in accuracy is observed with an increase in the overlap between the two subjects.

### 4.6. Comparison with Existing Studies

**Figure 11:**
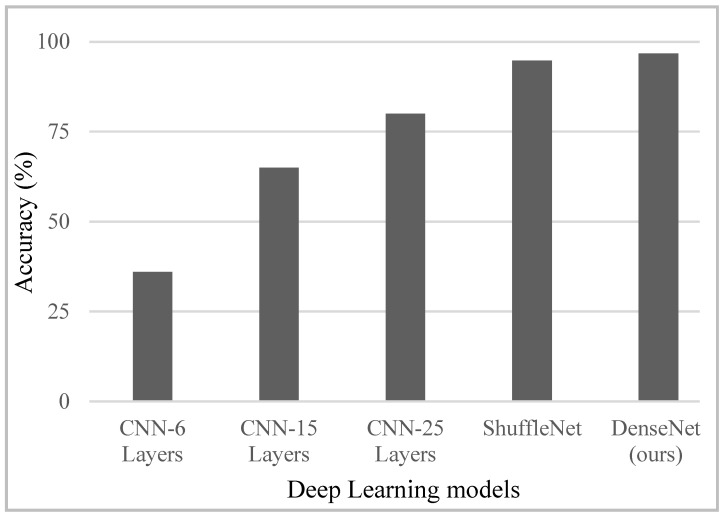
Classification accuracy of different deep learning models.

As stated earlier, the existing works often consider a single human target for posture evaluation [28,29,30,31,34]. Due to a lack of target segregation mechanism in the related studies, a direct comparison with the existing works cannot be performed. Nevertheless, in order to verify the effectiveness of the proposed framework based on target segregation followed by the DenseNet model, we compared several deep learning architectures on the generated dataset. Prominent convolution-based deep learning models were evaluated against the ten posture combinations shown earlier in Figure 5. The classification accuracy for each network is reported in Figure 11. A CNN comprising of 6, 15, and 25 layers resulted in 40%, 69%, and 73% accuracy, respectively. Note that in the two-human scenarios, the posture of both the participants must be correct. Another deep learning architecture named ShuffleNet [40] was also considered for evaluation, which yielded 90% classification accuracy. Contrary to this, DenseNet achieved 96% accuracy.

## 5. Conclusions and Further Work

This paper presents a two-person posture recognition framework using a FMCW radar-generated 3D point cloud and deep learning. Using a DBSCAN clustering approach, two targets are separated, and the point cloud of each person is converted into an image. A DesneNet framework is deployed to learn the features, followed by classification. Evaluation at different distances and angles suggests that unlike existing works, the proposed work is distance and angle-insensitive, and can recognize four postures from multiple humans simultaneously, with an average accuracy of 96%. Additional evaluation for the overlapping humans case suggests that for partial overlaps of 33% and 66%, the modified DBSCAN can recognize standing, sitting on chair, and lying down postures with an average success rate of above 90%.

This study considered four basic postures, named as standing, sitting on chair, sitting on floor, and lying down. Additional real-world postures can be included to imitate practical scenarios. Additionally, a lighter deep learning model for edge computing can be considered in future. For the case of overlapping human subjects, the missing point cloud data is not interpolated; instead, the targets are separated and fed to the deep learning model individually. The presented framework algorithm can accurately classify when there is a partial overlap between two humans separated by few centimeters. MIMO radar also monitors the depth information along with the horizontal and vertical angles. However, a strategy to mitigate the scenario of two closely located humans with no distance separation has not been devised so far. Such an approach could efficiently increase the robustness of multi-human posture detection works. Additionally, missing point interpolation could significantly increase the accuracy in evaluation of overlapping human subjects. 

## Figures and Tables

**Table 1:** Existing studies related to human posture classification (Tick represents if study possess the mentioned characteristics).

Study	Multiple Humans	Posture Combinations	Different Distances	Different Angles	Overlap Case
[24] (2014)	-	3	✓	-	-
[25] (2018)	-	3	-	-	-
[26] (2021)	-	6	-	-	-
[13] (2022)	-	6	-	✓	-
[27] (2022)	-	3	✓	✓	-
[28] (2022)	-	3	-	-	-
[34] (2022)	-	4	-	-	-
[29] (2023)	-	5	-	-	-
[30] (2023)	-	1	-	-	-
[31] (2024)	-	4	-	-	
[32] (2024)	-	6	-	-	-
[33] (2024)	-	3	-	-	-
[35] (2024)	-	3	-	-	-
[36] (2024)	-	4	✓	✓	-
Ours	✓	10	✓	✓	✓

**Table 2:** Radar parameters used for data acquisition.

Radar Parameter	Value / Description
Starting frequency	77 GHz
Total bandwidth span	3.3 GHz
Number of chirps	32
Frame rate	20 FPS
Number of frames	20
ADC samples per chirp	256
Number of Tx and Rx antennas	12 and 16
Azimuth and elevation antennas	86 and 4

**Table 3:** Evaluation of class-wise and mean accuracy, precision, recall, F1-score, specificity, and AUC values for the ten posture combinations in non-overlapping cases.

Scenario	Metric	1	2	3	4	5	6	7	8	9	10	Mean
Non-Overlap	Accuracy (%)	100	88.89	100	100	100	100	83.33	100	94.44	94.44	96.11
	Precision (%)	94.74	100	90	100	100	100	100	75	100	94.44	95.42
	Recall (%)	100	88.89	100	100	100	100	83.33	100	94.44	94.4	96.11
	F1-Score (%)	97.30	94.12	94.74	100	100	100	90.91	85.71	97.14	94.44	95.44
	Specificity (%)	99.35	100	98.69	100	100	100	100	98.15	100	99.35	99.55
	AUC	0.99	0.94	0.99	1	1	1	0.92	0.99	0.97	0.97	0.98

**Table 4:** Evaluation of class-wise and mean accuracy, precision, recall, F1-score, specificity, and AUC values for the combination of overlap and non-overlap cases.

Scenario	Metric	Both Standing	Standing + Sitting	Stand + Lie Down	Sit + Sit	Stand + Lie Down	Mean
Non-Overlap	Accuracy (%)	100	100	100	97.78	98.89	99.33
	Precision (%)	100	100	100	94.4	94.74	97.84
	Recall (%)	100	100	100	94.4	100	98.89
	F1-Score (%)	100	100	100	98.61	98.61	99.44
	Specificity (%)	100	100	100	98.61	98.61	99.44
	AUC	1.00	1.00	1.00	0.95	0.99	0.99
33% Overlap	Accuracy (%)	94.4	93.75	100	94.11	94.11	95.29
	Precision (%)	94.4	93.75	94.74	100	94.11	95.41
	Recall (%)	94.44	93.75	100	94.12	94.12	95.29
	F1-Score (%)	94.44	93.75	97.3	96.97	94.12	95.32
	Specificity (%)	98.53	98.57	98.53	100	95.55	98.84
	AUC	0.97	0.96	0.99	0.97	0.96	0.97
66% Overlap	Accuracy (%)	94.4	94.4	100	83.3	83.3	91.1
	Precision (%)	85	94.44	94.73	100	83.3	91.5
	Recall (%)	94.4	94.4	100	83.33	83.33	91.1
	F1-Score (%)	89.47	94.44	97.29	90.91	83.33	91.1
	Specificity (%)	95.83	98.61	98.61	100	95.83	97.8
	AUC	0.95	0.97	0.99	0.92	0.90	0.94

## Data Availability

The data are available on request.
